# Identification of a novel monocyte/macrophage-related gene signature for predicting survival and immune response in acute myeloid leukemia

**DOI:** 10.1038/s41598-024-64567-7

**Published:** 2024-06-18

**Authors:** Yun Zhan, Sixing Ma, Tianzhuo Zhang, Luxin Zhang, Peng Zhao, Xueying Yang, Min Liu, Weiwei Cheng, Ya Li, Jishi Wang

**Affiliations:** 1 Department of Hematology, Affiliated Hospital of Guizhou Medical University, Guiyang, 550004 People’s Republic of China; 2 Department of Clinical Medical School, Guizhou Medical University, Guiyang, 550004 People’s Republic of China; 3 Department of Vascular Surgery, Affiliated Hospital of Guizhou Medical University, Guiyang, 550004 People’s Republic of China; 4 Guizhou Province Institute of Hematology, Guizhou Province Hematopoietic Stem Cell Transplantation Center, Affiliated Hospital of Guizhou Medical University, Guiyang, 550004 People’s Republic of China

**Keywords:** Monocyte/macrophage, Tumor immune microenvironment, Immune response, AML, Cancer genetics, Tumour biomarkers

## Abstract

Acute myeloid leukemia (AML) is a heterogeneous hematological tumor with poor immunotherapy effect. This study was to develop a monocyte/macrophage-related prognostic risk score (MMrisk) and identify new therapeutic biomarkers for AML. We utilized differentially expressed genes (DEGs) in combination with single-cell RNA sequencing to identify monocyte/macrophage-related genes (MMGs). Eight genes were selected for the construction of a MMrisk model using univariate Cox regression analysis and LASSO regression analysis. We then validated the MMrisk on two GEO datasets. Lastly, we investigated the immunologic characteristics and advantages of immunotherapy and potential targeted drugs for MMrisk groups. Our study identified that the MMrisk is composed of eight MMGs, including HOPX, CSTB, MAP3K1, LGALS1, CFD, MXD1, CASP1 and BCL2A1. The low MMrisk group survived longer than high MMrisk group (P < 0.001). The high MMrisk group was positively correlated with B cells, plasma cells, CD4 memory cells, Mast cells, CAFs, monocytes, M2 macrophages, Endothelial, tumor mutation, and most immune checkpoints (PD1, Tim-3, CTLA4, LAG3). Furthermore, drug sensitivity analysis showed that AZD.2281, Axitinib, AUY922, ABT.888, and ATRA were effective in high-risk MM patients. Our research shows that MMrisk is a potential biomarker which is helpful to identify the molecular characteristics of AML immunology.

## Introduction

Acute myeloid leukemias are heterogeneous neoplasms characterized by uncontrolled proliferation and impaired differentiation of myeloid blasts, it has been increasing in incidence over the past few decades^1,2^. Even with intensive chemotherapy, risk stratification, and hematopoietic stem cell transplantation, relapses and/or progressive disease are the leading causes of death in patients with standard or high-risk AML^3^. The hypothesis of using the immune system to fight cancers, including leukemia, has been proposed for a long time, and over the past few years we have increased our understanding of the interaction between AML and immune cells, but so far there have been no major improvements from the lab to the clinic^4^. The immune microenvironment (TME) can impact ICI effectiveness, and only a few biomarkers can predict patient outcome accurately^5^. Hence, a novel strategy for sensitizing immune cell infiltration score(ICIs) in AML is required to develop predictive biomarkers for immunotherapy response.

In AML patients, the BM microenvironment is immunosuppressive, promoting tumor tolerance, immune escape, and disease progression^6^. It has been shown that AML cells and their surrounding hematopoietic microenvironment cross-talk extensively^7^. Monocytes/macrophages, also known as tumor-associated macrophages (TAMs), contribute significantly to the microenvironment in solid tumors^8^. The tumor microenvironment provides macrophages with a variety of factors that determine their polarization into M1 or M2 macrophages^9^. Recent studies have shown that mononuclear/macrophages in the AML bone marrow microenvironment are a plastic and heterogeneous population involved in AML survival and drug resistance^10^. It seems that APOE inhibits the proliferation of T cells in human monocytic AML cells and supports AML cell migration by activating LILRB4^11^. According to recent studies on mouse models, AML causes increased monocyte/macrophage counts in the bone marrow and spleen, supporting a protumorigenic microenvironment^8^. Other studies have shown that FOXC1 helps prevent monocyte/macrophage differentiation and enhances AML clonogenesis potential^12^. In particular, the AML microenvironment reflects the broad complexity of the prognostic significance of TAMs^8^. As a result, it is imperative to investigate the new strategy of targeting tumor monocytes/macrophages to alleviate the immunosuppressive TME and improve antitumor immunotherapy.

Our study aims to identify prognostic markers for AML associated with monocytes/macrophages that can be used to predict the effectiveness of conventional treatments and to suggest immunotherapies. Tumor Immune Single-Cell Hub (TISCH) dataset^13^ was used to identify the genes about monocytes/macrophages in AML based on single-cell RNA sequencing (ScRNA-seq). In this instance, we created the MMGs prognostic model (MMrisk). Additionally, we discussed the immunological characteristics of the MMrisk groups. The results of this study eventually revealed that MMrisk can predict outcome and success of immunotherapy in patients with AML. Our analysis suggests that MMrisk is a promising prognostic model targeting AML immune microenvironments.

## Materials and methods

### Clinical data source

An analysis of TCGA (https://portal.gdc.cancer.gov/) and GTEx (https://xenabrowser.net/datapages/) database was conducted on gene expression and clinical data. Among them, there were 150 samples of newly diagnosed patients, and 126 clinical samples were included for follow-up analysis after excluding patients with missing clinical data. Transcript counts were first normalized using the transcripts per million (TPM) method, the calculation method of TPM is to standardize the counts of each gene or transcript to the expression level of every million mapped transcripts through the STAR process, and then transformed using the log2 method. For validation, the GSE37642 (984 samples) and GSE12417 (405 samples) cohorts from Gene Expression Omnibus(GEO) database(https://www.ncbi.nlm.nih.gov/geo/) were used as independent cohorts. A monocytes/macrophages-related gene was found in three scRNA-seq datasets (GSE154109, GSE135851, and GSE116256) in the TISCH database^13^. In order to filter monocyte/ macrophage-related DEGs, P < 0.05 and |log2 FC|≥ 1 were applied. Additionally, Table 1 presents clinical characteristics of TCGA cohorts.

**Table 1:** Clinical features of AML patients in the TCGA database.

Characteristics	High	Low	P value
N	63	63	
Age, median (IQR)	61 (48, 69)	51 (38.5, 61.5)	0.002
Gender, n (%)			0.720
Male	36 (28.6%)	34 (27%)	
Female	27 (21.4%)	29 (23%)	
Bone Marrow_Blasts(%), median (IQR)	32 (8, 57)	37 (8.5, 68.5)	0.581
WBC_count(× 10^9^/L), median (IQR)	26 (7, 60.5)	11 (3, 33.5)	0.007
Cytogenetics risk, n (%)			0.003
Favorable	6 (4.8%)	22 (17.5%)	
Intermediate/normal	42 (33.3%)	30 (23.8%)	
Poor	15 (11.9%)	11 (8.7%)	
Risk score, median (IQR)	1.1749 (0.90406, 1.3756)	0.20079 (−0.13925, 0.47379)	< 0.001

### Characteristics of protein–protein interactions of MMGs

A network of protein–protein interactions (PPIs) was constructed using the STRING database^14^ and visualized using Cytoscape (version: 3.8.0)^15^ to determine each module's topology level.

### GO and KEGG analyses of MMGs

Gene Ontology (GO) and Kyoto Encyclopaedia of Genes and Genomes (KEGG) enrichment analyses were performed using the ClusterProfiler package^16^, which showed MMGs associated with the OS play key roles in AML.

### Establishment of risk prognostic model

MMGs with prognostic value were identified using the univariate Cox analysis of overall survival (OS). To avoid overfitting and determine the suitable number of monocyte/macrophage-related DEGs in model development (MMrisk), the LASSO regression analysis was conducted using the glmnet R package. Validation of the model was performed on training and test samples. We developed a risk prognosis model for the training and test groups.

$$\text{Risk score }= {\sum }_{i=1}^{n}(\text{MMGs expi}\times \text{coefi})$$

where n is the number of DEGs associated with OS prognosis that are MMGs-related, I is the ith monocyte/macrophage -related DEG, and coef is the regression coefficient. For each MMGs-related in the OS prognosis, the regression coefficient is multiplied by its expression, and then summed to determine the sample risk score^17^. The median value in the risk score is determined as the cut-off point, according to the median risk score, training, and test groups were separated into high- and low-risk groups.

### Enrichment analyses and the determination of differentially expressed genes (DEGs)

DEGs in high- and low-risk groups were identified using the limma software package^18^. A gene set enrichment analysis (GSEA) ^19^ was conducted in order to confirm the main enrichment pathways of the low-risk and high-risk cohorts. Nominal P value < 0.05 and FDR < 0.25 indicated significant enrichment.

### Validation of risk prognostic model

Various risk prognostic models were used for training and test groups, including risk curve analysis and survival analysis. Based on the risk prognosis model, survival status maps and risk heatmaps were created using R, along with OS prognosis MMGs and patient survival rates. ROC curves are plotted with its R package. To build survival curves, a survival program and a survminer are used. To determine if the risk score is an independent prognostic factor, univariate and multivariate COX regression models were used in R, along with the survival package.

### Analysis of immune signatures and function enrichment for MMrisk

In order to evaluate immune score, stromal score, and ESTIMATE score of each sample, the Estimation of Stromal and Immune cells in Malignant Tumors using Expression Data (ESTIMATE) method was used^20^. CIBERSORT” ^21^ were used to analysis the relationship between the ICI components.As a result, R package "ConsensusClusterPlus"^22^ implemented ICI-based hierarchical agglomerative clustering.

### Drug sensitivity and mutation analysis

Mutation annotation format (MAF) from the TCGA-LAML was generated using the "maftools" R package to identify the differences in somatic mutations of AML patients between high- and low-MMrisk groups^23,24^. The chemotherapeutic response of AML patients was assessed by genomics of drug sensitivity in cancer (GDSC)^25^. We utilized “pRRophetic” package^26^ to assess the chemotherapeutic response based on the 50% maximum inhibitory concentration.Based on the semi-inhibitory 146 concentration (IC50) values, we compared sensitivity of common chemotherapeutic agents used for treating AML between the two groups.

### Statistical analysis

R Studio and SPSS v.23.0 software (IBM Corp.) were used for all statistical analyses. In order to assess significant differences between the two groups, Student's t-tests were used for independent samples, whereas Spearman's correlation analysis was used for correlation analysis.

## Results

### An analysis of DEGs associated with MMGs in AML

**Figure 1:**
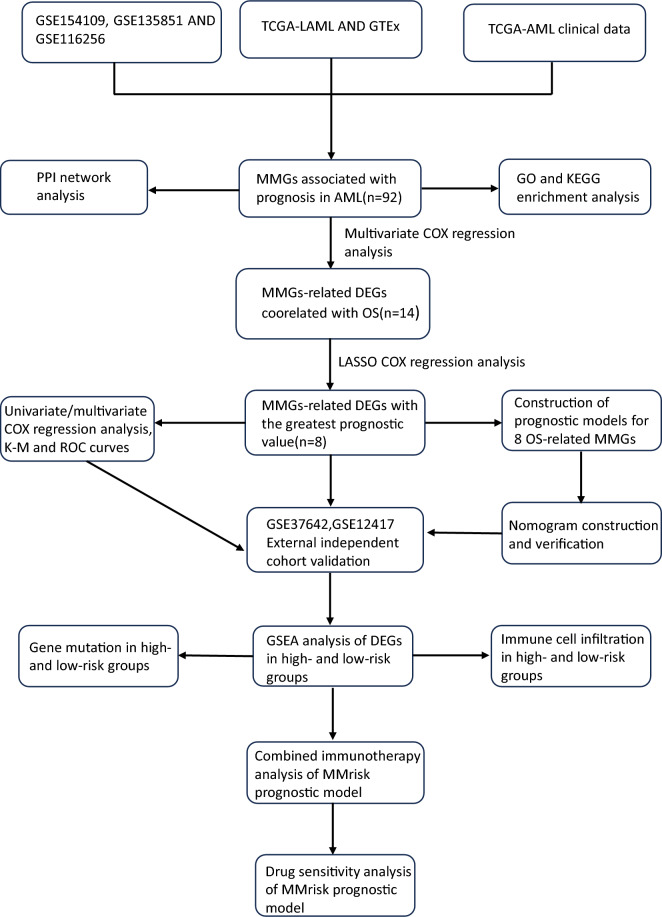
The flow chart of our research.

**Figure 2:**
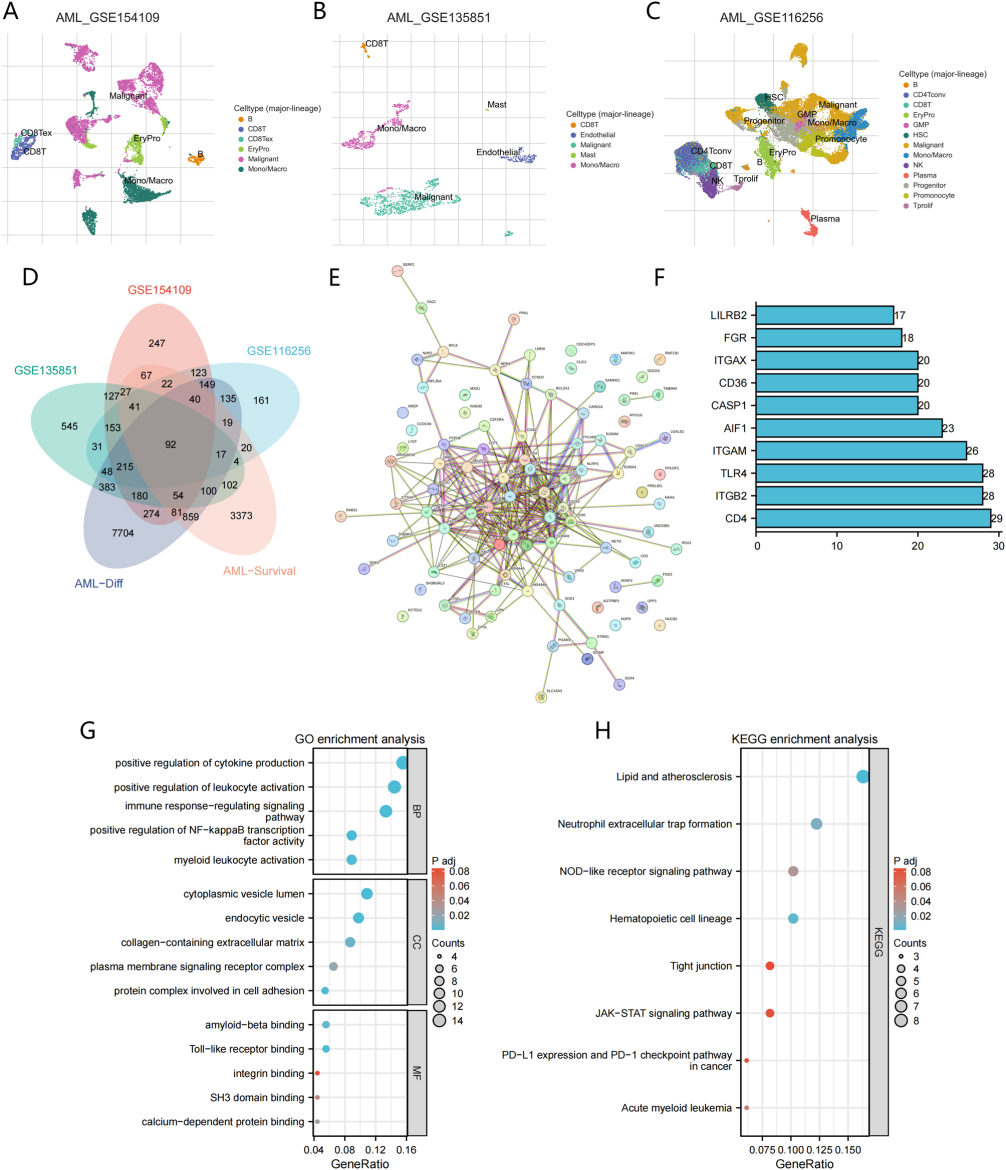
Identification of monocyte/macrophage-related DEGs. (**A**) The UMAP plot in GSE154109. (**B**) The UMAP plot in GSE135851. (**C**) The UMAP plot in GSE116256. (**D**) Venn plot overlay of three single-cell data sets with MMGs, TCGA-AML and GTEx DEGs and AML OS-related genes. (**E**) Network of protein–protein interactions among OS-related MMGs. (**F**) One-dimensional histogram of top10 key gene interaction of MMGs. (**G**) The bar plot showing OS-related MMGs by KEGG biological process. (**H**) The bar plot showing OS-related MMGs by GO biological process.

Figure 1 illustrates the general study workflow. TISCH is a rich single-cell database for studying the tumor microenvironment. In this study, we used the TISCH database to extract mononuclear/macrophage-associated differential genes from three AML single-cell datasets(GSE154109, GSE135851, and GSE116256)(Fig. 2A–C). Then we screened differentially expressed genes (log2FC > 1, P < 0.05) between AML and normal samples from TCGA and GTEx databases. At the same time, we screened genes related to AML’s OS from TCGA clinical sample data set, and obtained 92 monocyte/macrophage characteristic genes related to the prognosis of AML after crossing the above gene sets(Fig. 2D). Further, hub genes including CD4, ITGB2, TLR4, ITGAM and AIF1 were identified by protein–protein interaction network(Fig. 2E, F). In order to further explore the biological function of these genes, GO and KEGG pathway annotation analysis was performed. GO enrichment analysis showed that 92 MMGs were mainly related to positive regulation of cytokine production, ositive regulation of leukocyte activation, immune response-regulating signaling. KEGG analysis indicated that 92 MMGs were mainly enriched in Lipid and atherosclerosis, Neutrophil extracellular trap formation, NOD-like receptor signaling pathway, Hematopoietic cell lineage. These regulatory mechanisms of MMGs may play a key role in the pathogenesis of AML microenvironment.

### Establishment of OS-related MMGs prognostic model

**Figure 3:**
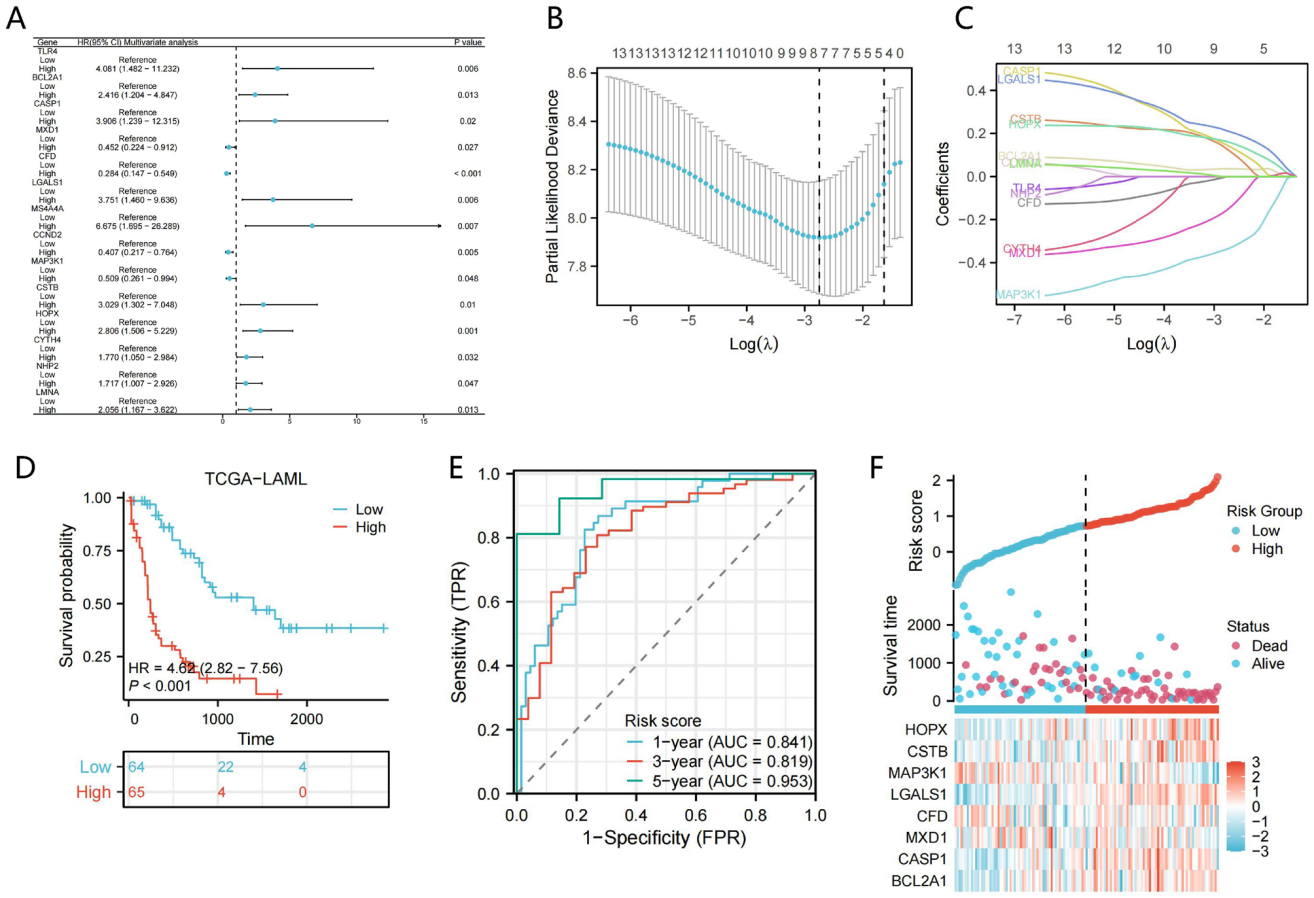
OS-related MMGs signature for prognosis construction. (**A**) Multivariate Cox regression analysis indicates remarkable relevance of 9 MMGs to OS. (**B,C**) A total of nine prognostic MMGs were used to construct the LASSO coefficient profiles, and the tuning parameter (λ) was calculated based on the minimum criteria for OS with ten-fold cross-validation. A best-fit profile was used to select eight genes. (**D**) Analysis of the TCGA cohort's high- and low-MMrisk groups using Kaplan–Meier survival curves. (**E**) An analysis of the ROC curves used for predicting OS in the TCGA cohort of AML patients at 1, 3 and 5 years. (**F**) Time distribution and survival status of patients with high and low MMrisks.

In order to construct a prognostic model of OS-related MMGs in AML, we included 92 genes in a single multivariate COX regression analysis to further screen the impact of these genes on the prognosis of AML., and 14 OS-related MMGs have been obtained(Fig. 3A). Among them, except MXD1, CFD, CCND2, MAP3K1, which are four protective genes with hazard ratio (HR) < 1, other genes are risk genes with HR > 1. To avoid potential over-fitting, LASSO-COX regression analysis was further used to screen key OS-related MMGs, eight MMGs, including HOPX, CSTB, MAP3K1, LGALS1, CFD, MXD1, CASP1, and BCL2A1 were identified to further construct monocyte/macrophage-associated gene prognostic signatures(Fig. 3B, C). MMrisks were calculated by extracting expression levels and LASSO coefficients of eight MMGs for each patient: MMrisk = (0.163 × HOPX expression) + (0.129 × CSTB expression) + (−0.338 × MAP3K1 expression) + (0.213 × LGALS1 expression) + ( −0.002 × CFD expression) + ( −0.157 × MXD1 expression) + (0.147 × CASP1 expression) + (0.036 × BCL2A1 expression). According to the risk score, two cohorts of patients were classified: the high-risk cohort and the low-risk cohort. Compared to patients with low MMrisk, AML patients with high MMrisk had more deaths and shorter survival times (Fig. 3D, F). ROC curves were performed at 1, 3 and 5 years to evaluate the predictive value of MMrisk. Figure 3E shows that the AUCs of MMrisk at 1, 3 and 5 years was 0.841, 0.819, and 0.953, respectively. According to these results, MMrisk has excellent diagnostic efficacy for predicting AML prognosis.

### The prognostic significance of OS-related MMGs in patients with AML

**Figure 4:**
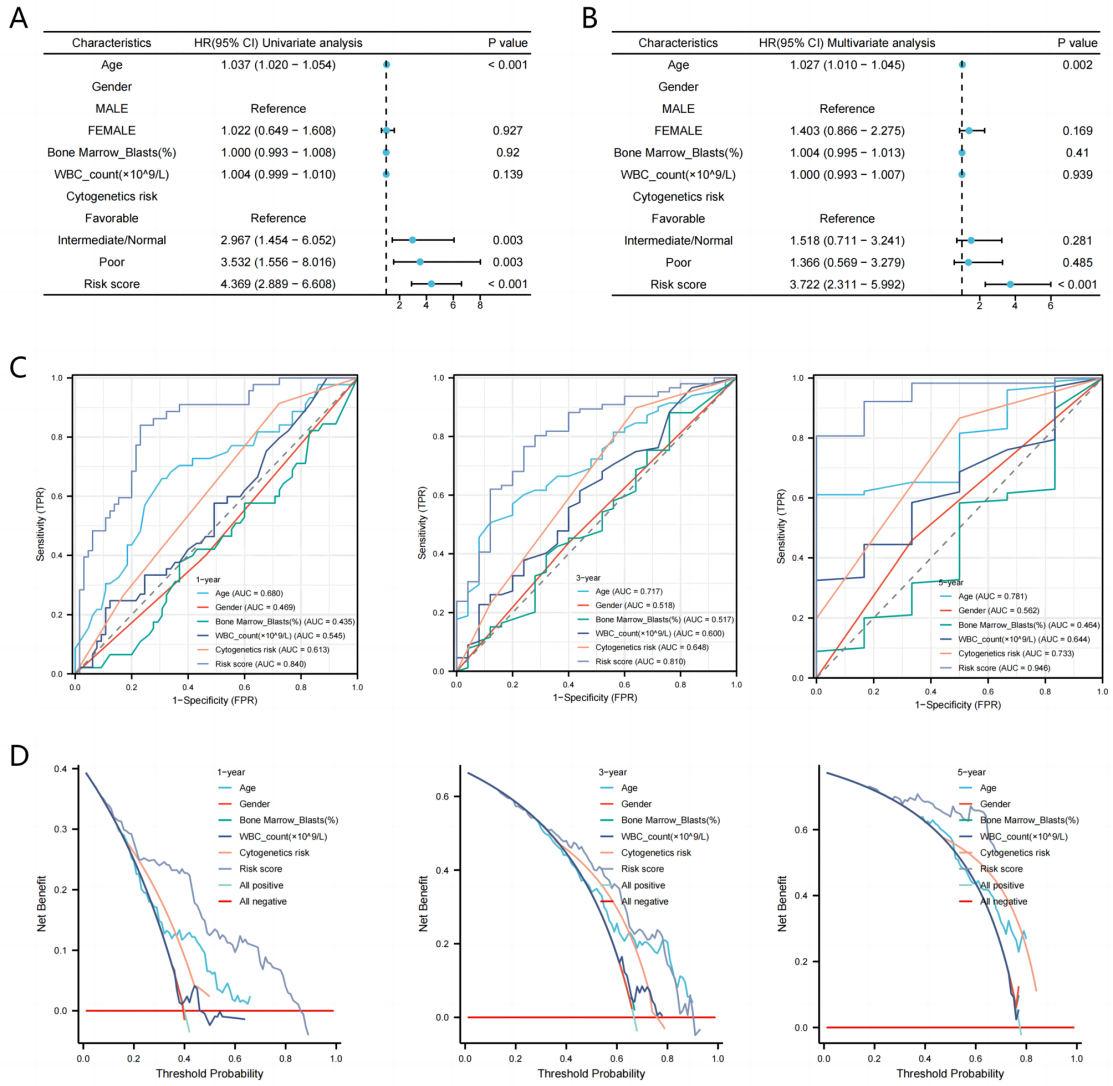
The prognostic significance of OS-related MMGs in patients with AML. (**A,B**) An independent prognostic factor affecting AML prognosis was found by univariate and multivariate Cox regression analyses based on MMGs. (**C**) Comparison of multiple clinical factors and MMrisk with ROC curve for predicting OS in 1 year, 3 years and 5 years. (**D**) DCA comparison chart of MMrisk and multi-clinical variables over 1 year, 3 years, and 5 years.

To evaluate the predictive ability of MMrisk for AML prognosis, We used univariate/multivariate COX regression to stratify the clinicopathological characteristics of AML patients, including age, sex, Bone Marrow Blasts, WBC_count, Cytogenetics risk, Favorable and Risk score. Univariate cox regression analysis showed that MMrisk, age, Cytogenetics risk of Intermediate/Normal, Poor were substantially related to the OS of AML (Fig. 4A). Furthermore, multivariable Cox regression analysis indicated that the MMrisk remained an independent prognostic indicator of MM after adjusting clinicopathological factors (Fig. 4B). In addition, clinical characteristics and risk scores were used to compare the predictive abilities of the MMrisk for patients with AML. Compared to clinical variables, the MMrisk had higher AUC values for predicting OS over 1, 3 and 5 years (Fig. 4C). The decision curve analysis(DCA) of 1-, 3-, and 5-year overall survival rate including MMrisk and other clinical factors revealed that the DCA value of MMrisk model was higher than other models(Fig. 4D). Collectively, these studies suggest that MMrisk may be reliable as an independent predictor for patients with AML and may also outperform other clinicopathological factor models in terms of predictive efficacy.

### Establishment and external verification of nomogram based on MMrisk

**Figure 5:**
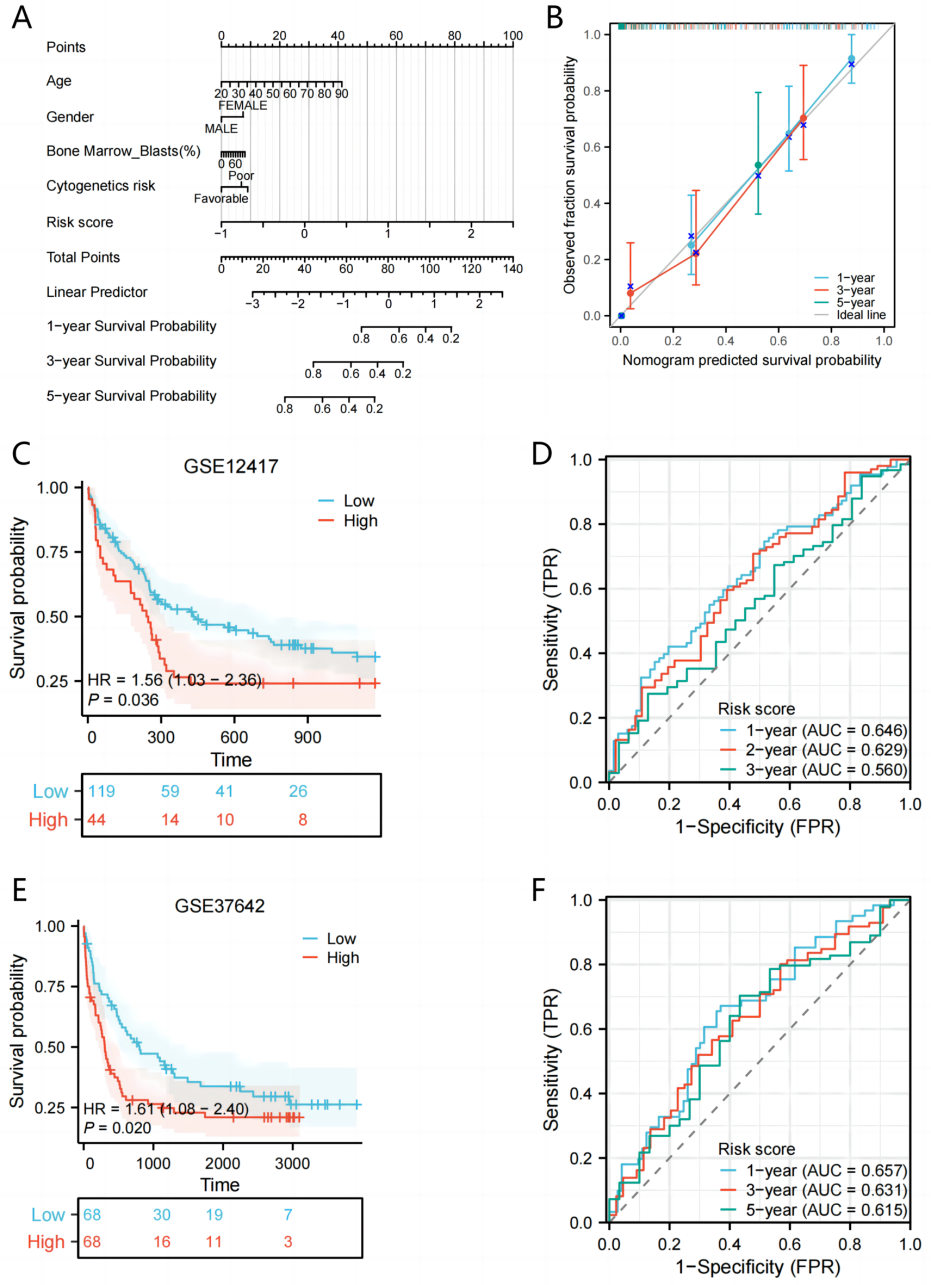
Establishment and external verification of Nomogram based on MMrisk. (**A**) nomogram combining MMGs with risk scores was developed to predict survival in 1, 3, and 5 years. (**B**) The calibration curves show the predictions for 1-, 3-, and 5-year overall survival for our nomogram. (**C**) Kaplan–Meier survival analysis of high- and low-MMrisk groups in the GSE12417. (**D**) ROC analysis for OS prediction including 1, 3, and 5 years of AML patients in the GSE12417. (**E**) Kaplan–Meier survival analysis of high- and low-MMrisk groups in the GSE37642. (**F**) ROC analysis for OS prediction including 1, 3, and 5 years of AML patients in the GSE37642.

In order to determine the signature in a highly accurate manner, a nomogram was plotted which included the MMrisk, age, gender, bone marrow-blasts, and cytogenetics risk (Fig. 5A). Based on calibration plots, it exhibited excellent concordance with OS predictions over1, 3, and 5 years (Fig. 5B). Like the TCGA results, the majority of new MMrisk identified in this analysis were negatively associated with the risk model in two test groups. In both the GSE12417 and GSE37642 test sets, AML patients with high MMrisk showed significantly shorter overall survival than patients with low MMrisk(Fig. 5C, E). Among them, the AUC values of 1 year, 3 years and 5 years in GSE12417 were 0.646, 0.629 and 0.650(Fig. 5D), respectively. In the GSE37642 dataset, the AUC values of 1 year, 3 years and 5 years were 0.657,0.631 and 0.615(Fig. 5F), respectively. Collectively, data above showed that the MMrisk was an independent predictor of OS in AML patients.

### Identification and enrichment of DEGs in MMrisk

**Figure 6:**
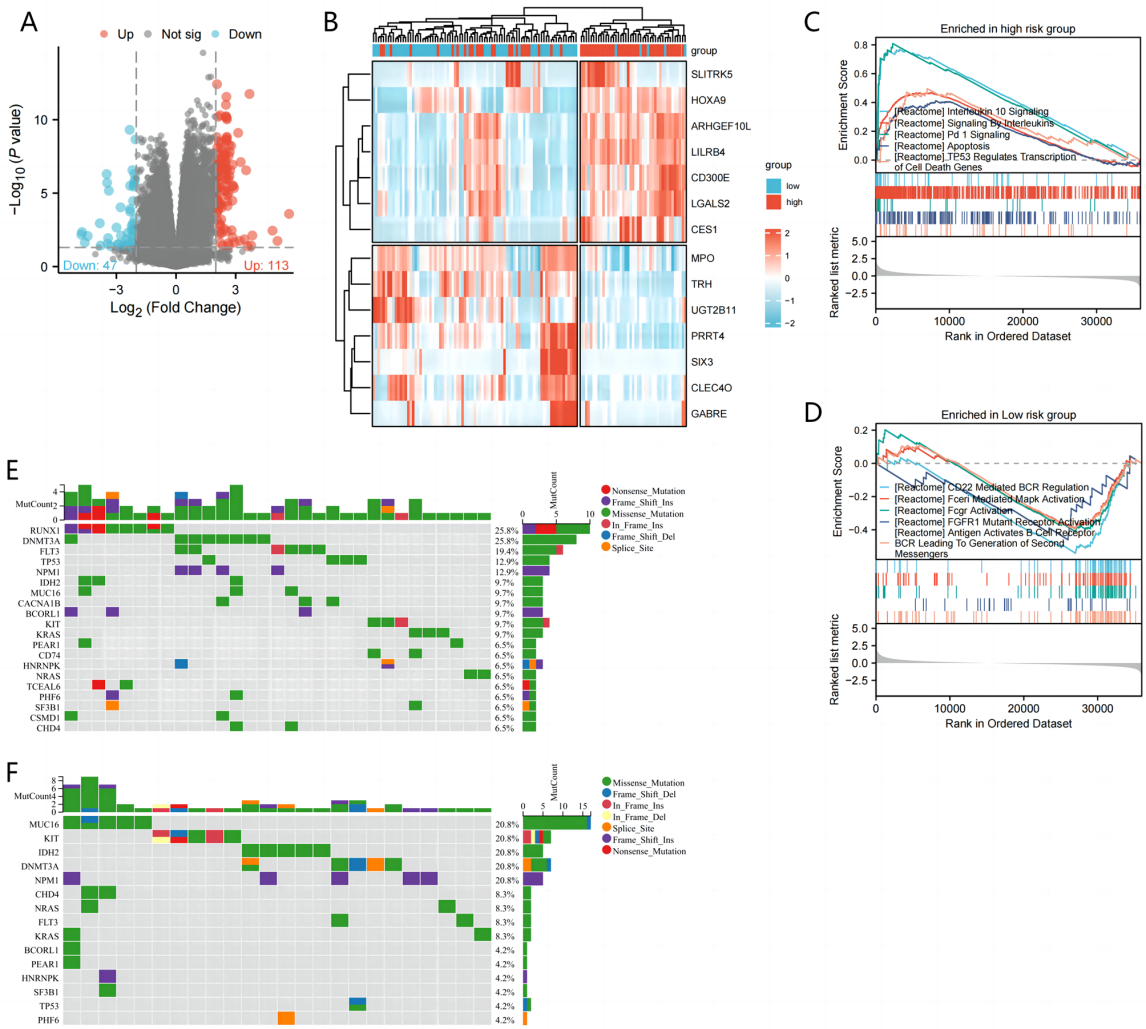
DEGs, GSEA and mutation analysis of high and low MMrisk groups. (**A**) DEGs of high and low MMrisk groups on a volcanic map. (**B**) Heat map of DEGs with high and low MMrisk groups. (**C**) Reactome gene sets enriched in the low MMrisk group. (**D**) Reactome gene sets enriched in the Low MMrisk group. (**E**) The mutation frequency of genes in the high MMrisk group. (**F**) The mutation frequency of genes in the low MMrisk group.

To further explore the differential gene and molecular function between patients with high and low MMrisk, we first screened a total of 1445 DEGs using limma software package, of which 917 genes were up-regulated and 528 genes were down-regulated (Fig. 6A, B). Further analysis of GSEA enrichment revealed that the Interleukin 10 Signaling, Signaling By Interleukins, PD-1 Signaling and Apoptosis pathways were significantly activated in the high MMrisk group(Fig. 6C), while CD22 Mediated BCR Regulation, Fceri Mediated Mapk Activation, Fcgr Activation and FGFR1 Mutant Receptor Activation pathways were significantly enriched in the low MMrisk group(Fig. 6D). To evaluate genomic characterization of MMrisk associated with AML, we made a visual analysis of somatic cell copy number alternation(SCNA) and mutation frequency in patients with TCGA-LAML. As shown in Fig. 6E, the mutation frequency of RUNX1, DNMT3A and FLT3 was the highest in the high MMrisk group, while the mutation frequency of MUC16, KIT, IDH2 and NPM1 was higher in the low MMrisk group (Fig. 6F). These results show that the high MMrisk group is more related to the immune regulation mechanism and has higher risk molecular genetic characteristics.

### A comparison of low and high MMrisk groups' immune characteristics

**Figure 7:**
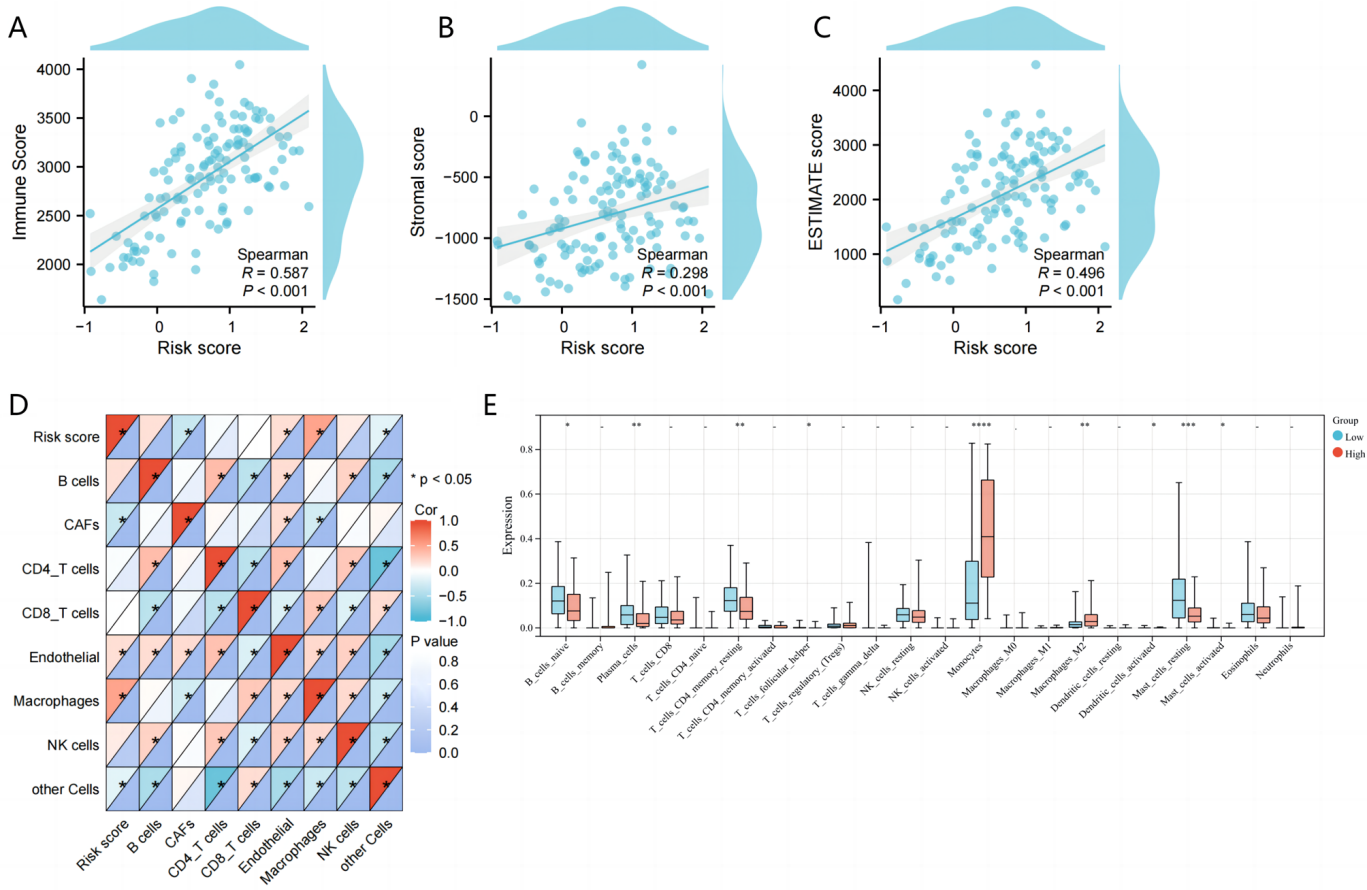
Identification of immune-related characteristics of MMrisk in AML. (**A**) Correlations of the MMrisk with immune score calculated by ESTIMATE algorithm. (**B**) Correlations of the MMrisk with stromal score calculated by ESTIMATE algorithm. (**C**) Correlations of the MMrisk with ESTIMATE score calculated by ESTIMATE algorithm. (**D**) EPIC correlation heat map shows the relationship between MMrisk and immune infiltration in AML. (**E**) The proportions of TME cells in high and low MMrisk groups by CIBERSORT analysis.

Progression of tumors is innately regulated by the tumor immune microenvironment^27^. In order to further explore the variation of immune characteristics between high and low MMrisk groups, the ESTIMATE algorithm was used to evaluate the immune score and stromal score of AML patients. We observed that the MMrisk was positively correlated with the immune score, stroma score and ESTIMATE score in the TCGA dataset (Fig. 7A–C). To analysis the association between MMrisk signature and immune cell infiltration in TME in the training set. CIBERSORT was used to indicate that Monocytes and Macrophages_M2 were activated in the high- MMrisk group, while the number of immune infiltrating cells such as B_cells_naive, Plasma_cells, T_cells_CD4_memory_resting and master _ cells _ resting increased significantly in the low- MMrisk group (Fig. 7E). The result of EPIC analysis showed that high MMrisk is positively correlated with Endothelial and Macrophages, and negatively correlated with CAFs (Fig. 7D). These results indicate that the regulatory role of monocyte/macrophage-related genes in AML microenvironment is more likely to cause strong immunosuppression.

**Figure 8:**
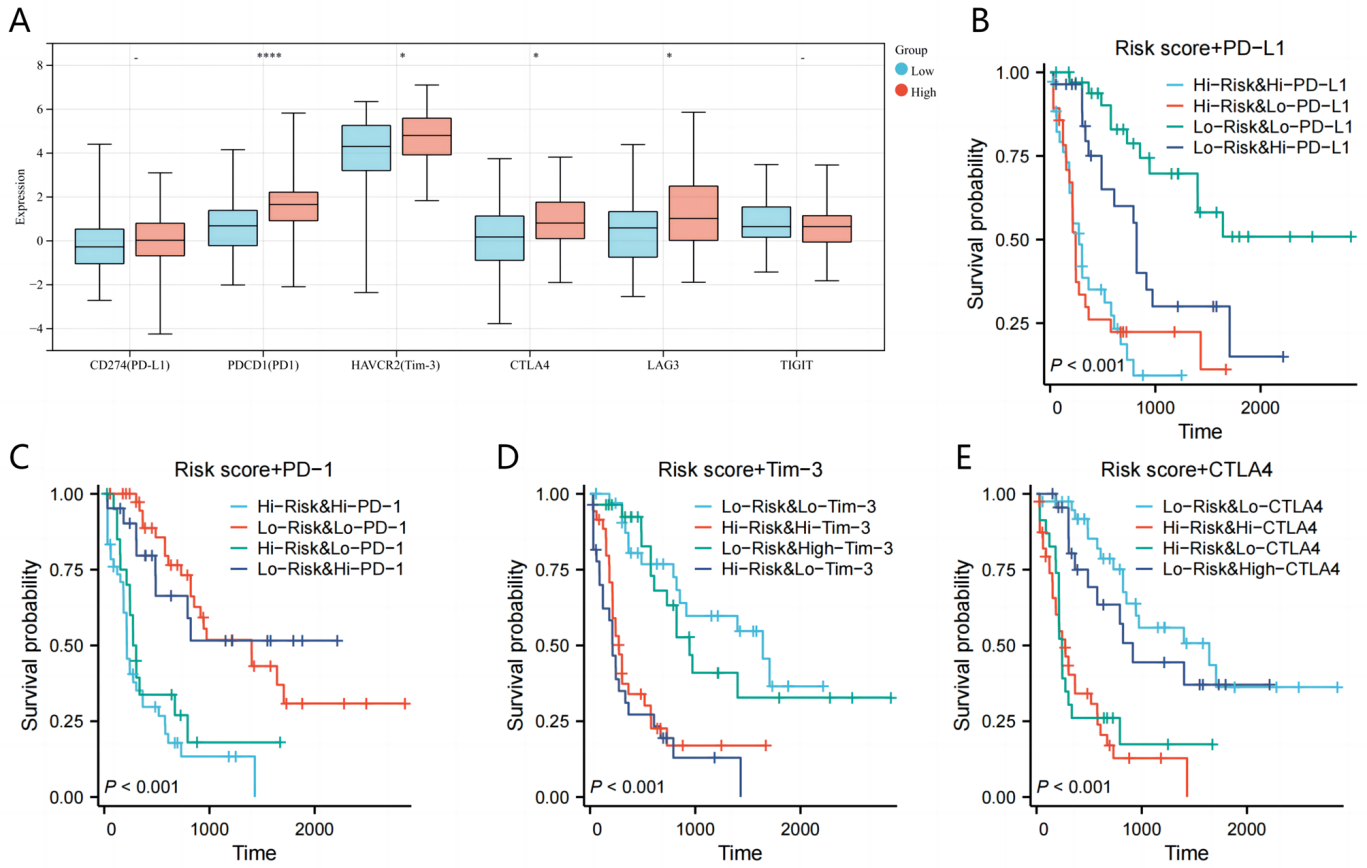
AML patients with MMrisk are predicted to respond to immune checkpoint inhibitors. (**A**) Expression of immune checkpoint in high and low MMrisk. (**B**) MMrisk and PD-L1 expression in four groups stratified by Kaplan–Meier survival curves. (**C**) MMrisk and PD-1 expression in four groups stratified by Kaplan–Meier survival curves. (**D**) MMrisk and HAVCR2 (TIM3) expression in four groups stratified by Kaplan–Meier survival curves. (**E**) MMrisk and CTLA4 expression in four groups stratified by Kaplan–Meier survival curves.

Apart from immune cells, TME includes immune checkpoint regulators as well as inflammatory mediators^28^. We examined the association between the MMrisk and immunotherapy response in AML to identify molecular mechanisms altering immunotherapy responsiveness. A higher level of PD-1, HAVCR2 (TIM3), CTLA4 and LAG3 expression was seen in the high MMrisk group compared to the low MMrisk group (Fig. 8A). K-M curve was used to analyze the prognosis of patients in high- and low-MMrisk groups combined with patients in immune high- and low- checkpoint expression groups. We found that whether combined with any of the above-mentioned immune checkpoints of PD-L1, PD-1, HAVCR2 (TIM3) and CTLA4, the prognosis and survival of the low MMrisk group combined with the low expression immune checkpoint group were significantly better than those of other groups (Fig. 8B–E). According to these results, MMrisk is a potentially useful biomarker for the prediction of immune combination therapy's response.

### Correlation analysis between MMrisk and drug sensitivity

**Figure 9:**
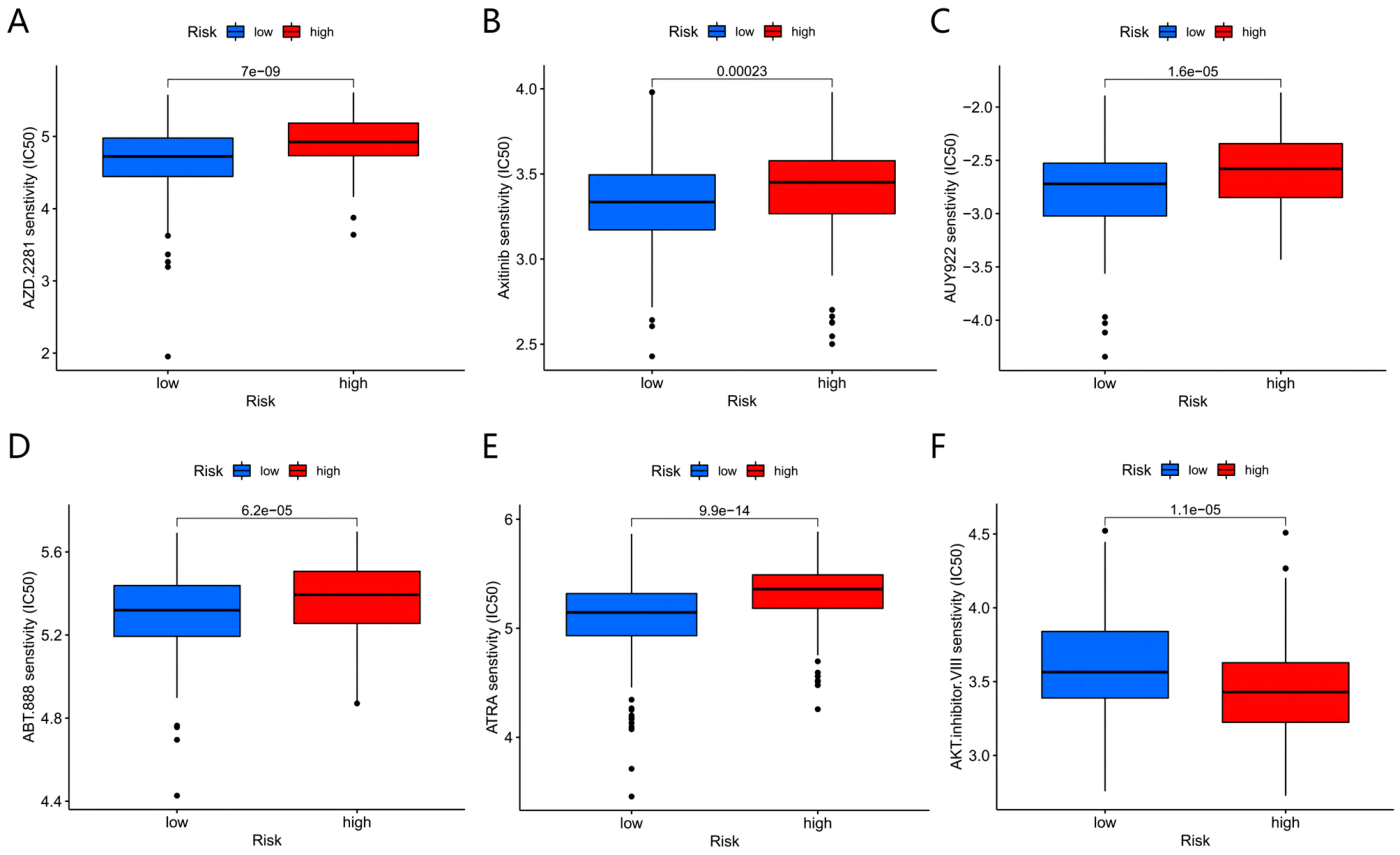
Evaluation of MMrisk and drug sensitivity. The IC50 of (**A**) AZD.2281(Olaparib), (**B**) Axitinib, (**C**) AUY922(Luminespib), (**D**) ABT.888(Veliparib), (**E**) ATRA and (**F**) AKT.inhibitor.VIII between low and high MMrisk groups.

Finally, in order to further identify potential drugs targeting monocytes/macrophages for AML, further, we compared the IC50 level of chemotherapeutic drugs in groups at low- and high- MMrisk. Results showed that the IC50 for the anti-cancer drugs AZD.2281(Olaparib), Axitinib, AUY922(Luminespib), ABT.888(Veliparib), and ATRA were higher in the high MMrisk group(Fig. 9A–E). Contrary to this, the IC50 for anticancer drugs such as AKT.inhibitor.VIII was higher in the low MMrisk group(Fig. 9F). These results suggest that AZD.2281(Olaparib), Axitinib, AUY 922(Luminespib), ABT.888(Veliparib) and ATRA have potential therapeutic effects on patients with high MMrisk.

## Discussion

There is no doubt that immunotherapy has emerged as a powerful clinical strategy for treating cancer. For AML patients, the immune environment of bone marrow has undergone profound changes, leading to the severity of the disease. Due to the complex interaction between AML cells and many components of their environment, based on the latest evidence of AML immunodeficiency, the treatment method of selective immune targeting AML cells is still facing challenges^29^. Researchers show that scRNA-seq technology can be used to investigate tumor heterogeneity and identify potential therapeutic targets by examining tumor heterogeneity and different cell subpopulations^30^.The purpose of this study was to investigate monocyte/macrophage-related gene expression in AML and identify a prognostic signature using the training cohort. TCGA test cohorts and GEO validation cohorts were used to evaluate the signature's predictability. Further, we found an increase in immune score, stromal score, immune cell infiltration, immune checkpoints, and somatic mutations in the high-risk MMrisk group. AML patients treated with targeted monocyte/macrophage combined with immune checkpoint can get better prognosis.

The prognostic signature was composed of eight genes related to monocytes/macrophages, including HOPX, CSTB, MAP3K1, LGALS1, CFD, MXD1, CASP1, and BCL2A1. It has been reported that the deletion of HOPX expression is very common in cancer, and it mainly acts as a tumor suppressor gene^31^. CSTB is associated with the pathogenesis of many malignant tumors. In pancreatic cancer, CSTB enhances the later stage of metastasis cascade by improving the formation of invasive pseudopodia and extravasation in vivo and in vitro^32^. In some studies, MAP3K1 was found to be an oncogene and to be associated with poor prognoses^33,34^. It has also been found to be a potential biomarker that can be targeted in mixed phenotype acute leukemia^35^. LGALS1 is closely related to AML. Some studies have shown that it is related to chemotherapy resistance and poor prognosis of AML patients^36^. The strategy for LGALS1 may be beneficial to AML patients^37^. CFD has been found to be a fat factor secreted from breast adipose tissue, which can enhance CSC characteristics in breast cancer^38^. CASP1 and BCL2A1 were also found to easily lead to poor prognosis and chemotherapy resistance in AML patients^39,40^. At present, MDX1 has not been reported to be directly related to AML, but some studies have found that MXD1 may be the therapeutic target of extrahepatic cholangiocarcinoma cells^41^. In addition, our study explored the expression patterns and prognosis of these genes, It seems likely that the signature genes identified in this study may provide potential molecular mechanisms for pursuing further clinical studies of AML.

We established the prognostic signatures of 8 monocyte/macrophage related genes in TCGA-AML data set, which were verified in GEO data set. Detection of the signature was consistent across both cohorts, indicating its robustness and reproducibility. A nomogram was also constructed to visualize and predict patients' survival rates at 1-, 3-, and 5-year. AUC, calibration plots, and DCA have all demonstrated a better predictive accuracy for the nomogram. Therefore, the nomogram can guide the establishment of personalized examination procedures for AML patients and promote the diagnosis and treatment effect.

TME plays a key role in anti-tumor reaction and can affect the prognosis of the disease^42^. Our study examined the correlation between MMrisk and TME. Firstly, we observed that MMrisk was positively correlated with immune score, stromal score, and ESTIMATE score. As well, the low-risk group was found to have a higher percentage of B_cells_naive, Plasma_cells, T_cells_CD4_memory_resting, and master_cells_resting levels of immune cells. Hence, these patients may be exhibiting relatively active antitumor immunity. Additionally, the ICIs have also been proposed as potential therapeutic targets for AML^43^. We found that the high-risk group showed high levels of expression of immune checkpoint genes (PD-1, HAVCR2 (TIM3), CTLA4 and LAG3). Meanwhile, low MMrisk combined with any of the above-mentioned immune checkpoint low expression groups can obtain the best survival prognosis. It indicates that AML patients in the low MMrisk group can benefit from immunotherapy targeting the current immune checkpoint. According to our findings, immunotherapy may be more effective in high MMrisk AML patients because of an increase in immune cell infiltration and immune response.

An analysis of drug sensitivity was conducted in different risk groups to better guide AML treatment. Our study compared the effects of six anticancer drugs, including AZD.2281(Olaparib), Axitinib, AUY922(Luminespib), ABT.888(Veliparib), ATRA and AKT.inhibitor.VIII among high-risk and low-risk patients. The results showed that the low MMrisk group was sensitive to AKT.inhibitor.VIII anticancer drugs, while the others were highly sensitive in the high MMrisk group which provided a reference for selecting chemotherapy drugs clinically. The follow-up study will investigate the clinical significance of these drugs in patients with AML.

While this study provided new insights into the development of immunotherapies to treat AML, it still had some limitations. First of all, it needs further in vivo and in vitro wet experiments to verify the exact functional phenotype of these monocyte/macrophage-related genes in AML. Second, all cohort studies were retrospective, requiring further validation with prospective cohort studies. Third limitation, only a small number of scRNA-seq samples and a limited amount of data were available in the public database, which may have led to potential bias in analyses of clinical and pathological parameters. Further verification will be necessary through the conduct of multi-center, large-sample, prospective double-blind trials and multiomics verification.

## Conclusions

In summary, our study combined scRNA-seq and bulk RNA-seq information to develop a novel prognostic signature that includes 8 monocyte/macrophage related genes. Additionally, TIME and immune-related pathways, as well as drug sensitivity, were significantly associated with the MMrisk. Targeting monocytes and macrophages in the TME might be beneficial for enhancing anti immune checkpoint inhibition and prognosis prediction in AML.

## Data Availability

Online repositories have provided the datasets in this study. The article includes the name of the repository/repositories and accession number(s).
